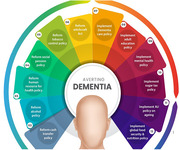# Economics of Dementia Prevention: Why the 2023 World Health Global Guidelines on Dementia May Not Reduce the Dementia Epidemiology in Developing Countries

**DOI:** 10.1002/alz.094651

**Published:** 2025-01-09

**Authors:** Cyprian M Mostert

**Affiliations:** ^1^ 1 Aga Khan University, Brain and Mind Institute, 3rd Parklands Avenue, Nairobi, Kenya, Nairobi, Nairobi Kenya

## Abstract

**Background:**

In 2023, the World Health Organization (WHO) presented the Mental Health Gap Action Programmes to avert Dementia. In this publication, the WHO presented a high certainty that physical activity interventions prevent Dementia. The organization presented low levels of certainty that psychosocial interventions, non‐pharmaceutical interventions, depression, and anxiety treatments are effective for Dementia prevention in low‐income and middle‐income countries (LMICs). This policy paper utilizes Global evidence, Continental perspective, Regional insight, and County outlook and presents the potential global policies that can avert Dementia, which were omitted in the 2023 WHO report.

**Method:**

This review uses World Bank, World Health Organization, and United Nations datasets. We also comprehensively searched the medical literature for published articles on social and commercial determinants of Dementia.

**Result:**

We demonstrate that the current WHO report does not comprehensively present policies that may yield positive returns in averting Dementia for developing countries. Physical activity is high in LMICs and may do little to prevent Dementia. Countries need to reduce poverty, hunger, tobacco consumption, alcohol consumption, sugar consumption, and other substances as crucial risk factors that drive Dementia outcomes in LMICs. Other policies that may improve Dementia epidemiology are welfare programs presented in Figure 1. These policies have robust chance of averting sixty percent of the Dementia cases.

**Conclusion:**

There is a significant risk of failing to improve Dementia outcomes if the current 2023 WHO report is not amended. Policymakers must address both the social and commercial determinants factors of Dementia as countries seek to achieve healthy brain aging.